# Recombinant Antimicrobial Peptide OaBac5mini Alleviates Inflammation in Pullorum Disease Chicks by Modulating TLR4/MyD88/NF-κB Pathway

**DOI:** 10.3390/ani13091515

**Published:** 2023-04-30

**Authors:** Shanshan Shen, Fei Ren, Junping He, Jie Wang, Yawei Sun, Jianhe Hu

**Affiliations:** 1 Henan Institute of Science and Technology, College of Animal Science and Veterinary Medicine, Xinxiang 453003, China; 2 College of Veterinary Medicine, Shanxi Agricultural University, Taigu 030801, China

**Keywords:** pullorum disease, *S.* Pullorum, antimicrobial peptides, recombinant expression, innate immunity

## Abstract

**Simple Summary:**

Pullorum disease (PD), caused by *Salmonella enterica* serovar Pullorum (*S*. Pullorum), is the most consequential poultry disease in countries with a developing poultry industry, which poses a considerable economic burden on the poultry industry. Antimicrobial peptides are key components of the innate immune system and are next-generation antibiotic agents with broad antimicrobial spectrum, low resistance, and low cytotoxicity. The aim of the present study was to generate a recombinant antimicrobial peptide, OaBac5mini, and explore its efficacy on PD. In this study, 1-day-old chicks were orally challenged with *S*. Pullorum to establish PD models. *S*. Pullorum markedly increased the organ indexes of the heart, liver, spleen, and kidney; induced histopathological changes in multiple organs; and impaired the innate immunity through the TLR4/MyD88/NF-κB pathway. Recombinant OaBac5mini was generated by an *Escherichia coli* recombinant expression system and exhibited strong antibacterial activity in *S*. Pullorum-challenged chicks. It decreased the organ bacterial loads in the liver and spleen, ameliorated the organ indexes and histopathological changes of chicks with PD, and reduced the expression of pro-inflammatory cytokines by modulating the innate immunity through the TLR4/MyD88/NF-κB pathway. These findings reveal the in vivo antibacterial activity of recombinant OaBac5mini against *S*. Pullorum and demonstrate its therapeutic potential as an antibiotic agent for PD.

**Abstract:**

Pullorum disease (PD), caused by *Salmonella* Pullorum (*S*. Pullorum), is a serious threat to the poultry industry worldwide. Antimicrobial peptides (AMPs) have drawn extensive attention as new-generation antibiotics because of their broad antimicrobial spectrum, low resistance, and low cytotoxicity. AMP OaBac5mini exhibits strong antibacterial activity against Gram-negative bacteria, but its efficacy and anti-inflammatory effects on chicks with PD remain unclear. The aim of this study was to generate recombinant OaBac5mini via the *Escherichia coli* (*E*. *coli*) recombinant expression system and evaluate its antibacterial effect against *S*. Pullorum in vitro and in vivo. Real-time cellular analysis (RTCA) results showed that recombinant OaBac5mini exhibited no cytotoxicity on IPEC-J2 and RAW 264.7 cells and significantly alleviated the drop in the cell index of *S*. Pullorum-infected cells (*p* < 0.0001). In the chick model of PD, recombinant OaBac5mini significantly attenuated the increase in organ indexes (heart, liver, spleen, and kidney) and bacterial loads (liver and spleen) induced by *S*. Pullorum. Histopathology examination showed that recombinant OaBac5mini ameliorated histopathological changes and inflammation in chicks with PD, including impaired epithelium of duodenal villi, infiltration of pseudoacidophilic granulocytes in the cecum and bursa of Fabricius, congested blood clots and increased macrophages in the liver, and increased lymphoid nodule and B lymphocytes in the spleen. Western blot and quantitative real-time PCR (qRT-PCR) results indicated that recombinant OaBac5mini alleviated inflammation by modulating innate immunity through the TLR4/MyD88/NF-κB pathway and by suppressing the expression of pro-inflammatory cytokines. These results suggested that recombinant OaBac5mini has good potential as a clinical substitute for antibiotics in PD intervention.

## 1. Introduction

Pullorum disease (PD) is caused by the serotype *Salmonella enterica* serovar Pullorum (*S*. Pullorum) and is one of the most important poultry diseases listed by the World Organization for Animal Health as it creates considerable economic burden in the poultry industry; it is most common in countries with developing poultry industries [1,2,3]. *S*. Pullorum is a serious threat to the poultry industry as it contributes to the high mortality rate of embryos and chicks [4] and is spread both vertically through the reproductive tract and horizontally from contaminated environments [5]. Most adult chickens infected with *Salmonella* are usually recessive in the absence of clinical signs and symptoms, but the pathogens can exist in the intestine, ovary, and eggshell surface [6], which might cause foodborne or zoonotic diseases [7].

The widespread attention to foodborne and zoonotic diseases is due to the increasing spread of resistant or multiresistant *Salmonella* strains resulting from the antibiotics used in the livestock industry [8,9]. Antibiotics used as feed supplements for growth enhancement in livestock were banned by the European Union Commission, U.S. Food and Drug Administration, and China in 2006, 2017, and 2020, respectively, to reduce the increasing risk of resistance development [10,11]. A potent alternative—antimicrobial peptides (AMPs), which are key components of the innate immune system—has drawn extensive attention because of its broad antimicrobial spectrum, low cytotoxicity, and low antimicrobial resistance brought by its unique bactericidal mechanism [12]. Cathelicidins are one of the most characterized AMP families; they exhibit strong antibacterial activity against Gram-negative bacteria with remarkably low cytotoxicity [13]. Bac5 is the first characterized member of cathelicidins; it has been identified in cattle, sheep, and goats [14]. OaBac5 is a Bac5 homolog derived from sheep neutrophils and is a linear proline-rich antibacterial peptide (PrAMP) with 51 residues [15]. Its truncated fragment, OaBac5mini, is a linear PrAMP consisting of 24 residues with eight net positive charges [16] that exhibits strong antibacterial activity against *S. enterica* serovar Typhimurium in vitro (minimum inhibitory concentration is 0.5 μg/mL) [17]. However, the antibacterial activities and mechanisms of OaBac5mini and its recombinant protein products against *S*. Pullorum in vivo remain unclear. Recombinant expression is the most efficient method for large-scale peptide production, offering substantial amounts of highly purified peptides to support both basic research and clinical trials [18]. In this study, we produced a fusion protein consisting of OaBac5mini and interferon (IFN) using a recombinant protein expression system in *Escherichia coli* (*E. coli*), which is the most commonly used host. This approach minimized toxicity to *E. coli* and allowed us to investigate the therapeutic potential of the fusion protein for PD, providing new insights for the production of recombinant AMPs and the development of PD treatment strategies.

## 2. Materials and Methods

### 2.1. Recombinant Expression of IFN_OaBac5mini

The recombinant expression vector pET-32a::IFN_OaBac5mini was designed as shown in Figure S1. In brief, IFN_OaBac5mini fragments containing an enterokinase site were amplified by three-step polymerase chain reaction (PCR) with PrimeSTAR Max Premix (TaKaRa, Kyoto, Japan). For the first step of PCR, pET28a-SUMO::IFN + PG plasmid was used as the template, and OaBacR1 containing restriction endonuclease sites was used as the primer (Forward: 5′-CCAGAGGTCAAGCCAGAAGT-3′ and Reverse: 5′-CGGCGGACGACGAATCGGCGGACGAAACCTCTTGTCGTCGTCGTCGCCT-3′). The PCR products were purified using E.Z.N.A^®^ Gel Extraction Kit (Omega, Norcross, GA, USA) and used as the template for the second step of PCR with the following primers: OaBacR2 (Forward: 5′-CCAGAGGTCAAGCCAGAAGT-3′ and Reverse: 5′-CGAAACGGAGGACGAAAAGGCGGACGAATCGGCGGACGACGAATCGGCGG-3′). The final PCR step was performed using the gel-purified PCR products from the second step of PCR with the following primers: OaBacR3 (Forward: 5′-CCAGAGGTCAAGCCAGAAGT-3′ and Reverse: 5′-TTCTCGAGTTAGCGCACCGGCGGGCGAAACGGAGGACGAAAAG-3′). The final-step PCR fragments were purified and digested with *Bam*HI (Thermo Fisher Scientific, Waltham, MA, USA) and *Xho*I (Thermo Fisher Scientific, USA). The plasmid pET-32a (+) was digested with the same restriction endonucleases and purified. Then, the linear vector (5860 bp) and IFN_OaBac5mini fragment (612 bp) were ligated with T4 ligase (Thermo Fisher Scientific, USA) overnight at 4 °C, repurified, and transferred into *E. coli* DH5α by electroporation (GenePulser Xcell PA-400, Bio-Rad, Hercules, CA, USA; time constant protocol: 1800 V voltage, 3 ms TC, 1mm cuvette). The transformants were selected on Luria–Bertani agar (Solarbio, Beijing, China) plates containing 60 μg/mL ampicillin (China National Institute for Drug and Biological Products Control, Beijing, China) at 37 °C. The inserted sequence was amplified from the ampicillin-resistant strains using primers (Forward: 5′-ACTGGATGAAATCGCTGACG-3′, Reverse: 5′-CCGAGATAGGGTTGAGTGTTGT-3′, 1492 bp) and sequenced by Sangon Biotech Co. (Shanghai, China).

The recombinant expression vector, pET-32a::IFN_OaBac5mini, was extracted from *E. coli* DH5α and electroporated into *E. coli* BL21. The positive strain was cultured in 2 × YT broth (Solarbio, Beijing, China) containing 80 μg/mL ampicillin and induced by 0.4 mM isopropyl ß-D-1-thiogalactopyranoside (IPTG, Solarbio, Beijing, China) for 14 h at 18 °C. The bacteria were centrifuged and resuspended with phosphate buffered saline (PBS, pH 8.0), followed by ultrasonic breaking using low-temperature ultra high-pressure continuous flow cell crusher JN-10C (Juneng Nano&Bio, Guangzhou, China) at 1800 bar pressure 4 °C and centrifuging. The inclusion body (IB) was renatured and purified with NI-NTA pre-packed gravity column (Sangon, Shanghai, China) following the instructions of the manufacturer and Chen et al. [19]. Then, the expressed recombinant AMP was collected and analyzed using sodium dodecyl sulfate–polyacrylamide gel electrophoresis (SDS-PAGE) and BCA kit (BioTeke, Wuxi, China) to determine its purity and concentration. Finally, IFN_OaBac5mini was digested with recombinant enterokinase (Beyotime, Shanghai, China) to obtain recombinant OaBac5mini.

### 2.2. Real-Time Cell Assay

Real-time cellular analysis (RTCA) was conducted to evaluate the cytotoxicity of the digested IFN_OaBac5mini. Intestinal porcine enterocytes (IPEC-J2) and macrophage cells (RAW 264.7) were cultured in high-glucose Dulbecco’s modified Eagle’s medium (DMEM; HyClone, Logan, UT, USA) supplemented with 10% fetal bovine serum (FBS; Biological Industries, Haemek, Israel) and 1% penicillin–streptomycin (Solarbio, Beijing, China) at 37 °C with 5% CO_2_. Cells were seeded in 16-well E-plates (xCELLigence, ACEA biosciences, San Diego, CA, USA). The next day, DMEM was replaced by fresh complete high-glucose DMEM containing different concentrations of digested IFN_OaBac5mini (25–400 μg/mL). Cell index, which is used to assess proliferation and cell viability [20,21], was monitored using the xCELLigence RTCA DP System (xCELLigence, ACEA Biosciences, San Diego, CA, USA) for 40 h at 15 min intervals and normalized by RTCA software 2.0 (xCELLigence, ACEA biosciences, San Diego, CA, USA).

RTCA monitoring was conducted using IPEC-J2 and RAW 264.7 cells to evaluate the protective effect of recombinant OaBac5mini on small intestinal epithelial cells and macrophages against *S*. Pullorum. In brief, cells were seeded in 16-well E-plates and rinsed with sterile PBS on the next day. The digested IFN_OaBac5mini (final concentration is 1 mg/mL for better antibacterial effect) and *S*. Pullorum CVCC 530 (National Center for Veterinary Culture Collection, Wuhan, China; the optimal multiplicity of infection (MOI) is 10:1) were mixed with high-glucose DMEM (without penicillin–streptomycin) alone or together and then added to each well. Monitoring was conducted for 35 h, and the cell index was normalized.

### 2.3. Total RNA Extraction and qRT-PCR

RAW 264.7 cells were seeded in a 6-well plate, cultured overnight, and then rinsed with PBS. High-glucose DMEM (without penicillin–streptomycin) containing digested IFN_OaBac5mini and/or *S*. Pullorum (MOI is 10:1) was added. Total RNA was extracted using TRIzol reagent (Ambion, Austin, TX, USA) following the manufacturer’s instructions and analyzed on NanoDrop 2000 (Thermo Scientific, Waltham, MA, USA) to determine the concentration. Quality was assessed by optical density 260/280 ratio and 1% agarose gel electrophoresis, and then cDNA was synthesized using EasyScript One-Step gDNA Removal and cDNA Synthesis SuperMix (TransGen Biotech, Beijing, China) with 1 μg of each RNA sample. Quantitative real-time PCR (qRT-PCR) was performed in triplicate using the following primers: *TNF-α* (Forward: 5′-CCCAGACCCTCACACTCAGATCATC-3′ and Reverse: 5′-GTTGGTTGTCTTTGAGATCCATGCC-3′), *IL-1β* (Forward: 5′-GAAATGCCACCTTTTGACAGTG-3′ and Reverse: 5′-TGGATGCTCTCATCAGGACAG-3′), *IL-6* (Forward: 5′-GGAGTCACAGAAGGAGTGGCTAAG-3′ and Reverse: 5′-AGTGAGGAATGTCCACAAACTGATA-3′), *IL-12* (Forward: 5′-TGGTTTGCCATCGTTTTGCTG-3′ and Reverse: 5′-ACAGGTGAGGTTCACTGTTTCT-3′) and reference gene *GAPDH* (Forward: 5′-AGGTCGGTGTGAACGGATTTG-3′ and Reverse: 5′-GGGGTCGTTGATGGCAACA-3′). The reactions were carried out with the 2× Universal SYBR Green Fast qPCR Mix (ABclonal, Prinzenallee, Germany) and run in the QuantStudio 5 Real-time PCR Instrument (Thermo Fisher Scientific, USA) under the following conditions: initial denaturing at 95 °C for 30 s, 40 cycles at 95 °C for 10 s, annealing at 58 °C for 20 s, and 72 °C for 20 s. Data were analyzed by the 2^−ΔΔCt^ method [22], and the transcriptional level of each gene was normalized to *GAPDH* level.

### 2.4. Animal Experiment Design

A total of 120 1-day-old laying hens were obtained by hatching specific pathogen-free (SPF) chicken embryos in our laboratory. The hens were randomly divided into four groups: control group (Control), *S*. Pullorum CVCC 530 infection group (*S*. Pullorum), recombinant OaBac5mini group (IFN_OaBac5mini), and *S*. Pullorum and recombinant OaBac5mini treatment group (*S*. P + IFN_OaBac5mini). Each group had 10 chicks, and three independent repeats were performed. The *S*. Pullorum CVCC 530 culture was centrifuged and resuspended with aseptic PBS into 1 × 10^9^ CFU/mL. For each 1-day-old chick in the *S*. Pullorum and *S*. P + IFN_OaBac5mini groups, 100 μL of bacterial suspension was administered orally for three consecutive days. After that, aseptic PBS was orally administered to each chick in the control group and *S*. Pullorum group for seven successive days, and each chick in the *S*. P + IFN_OaBac5mini group was given 100 μL of aseptic PBS containing 2 mg/mL digested IFN_OaBac5mini for seven successive days. After the 7-day therapeutic trial, the chicks were sacrificed humanely for the subsequent experiments.

### 2.5. Organ Index Determination

The heart, liver, spleen, and kidney were collected, washed with aseptic PBS, and weighed after the residual water was removed using filter paper. Organ index was calculated using the formula:

Organ index = Organ weight (g)/Body weight (kg).

### 2.6. Organ Bacterial Load Detection

The liver and spleen were aseptically collected, weighed, and homogenized in 1 mL of aseptic PBS using Tissuelyser (Hoder, Beijing, China) at 75 Hz and 4 °C for 5 min. The tissue homogenate was diluted gradiently by PBS, and 100 μL of dilutions were plated on xylose lysine desoxycholate agar (Hopebio, Qingdao, China) selective plates and cultured overnight at 37 °C to count the number of colonies. Organ bacterial load was calculated as follows:

Organ Bacterial Load = Number of colonies (CFU)/Organ weight (g).

### 2.7. Histopathology Examination

The duodenum, cecum, liver, spleen, and bursa of Fabricius were fixed with 4% paraformaldehyde for 24 h, dehydrated with gradient ethanol, clarified with xylene, and dipped in paraffin. The paraffin blocks were sliced into 4–6 μm-thick sections followed by conventional hematoxylin–eosin (HE) staining for histopathological examination.

### 2.8. Western Blot Assays

Total proteins of the spleen were extracted using radioimmunoprecipitation assay (RIPA; Solarbio, Beijing, China) buffer with 1% protease inhibitors (Boster, Wuhan, China) and 1% phosphatase inhibitors (Cowin Bio., Taizhou, China), and then qualified by bicinchoninic acid (BCA) kit (BioTeke, Wuxi, China) to determine their concentrations. Equal amounts of protein for each sample were separated in 10% SDS-PAGE and transferred onto polyvinylidene fluoride (PVDF) membranes (0.2 μm; Merk Millipore Ltd., Darmstadt, Germany). The PVDF membranes were blocked with 5% bovine serum albumin (Solarbio, Beijing, China) at room temperature for 1 h and then incubated overnight with the primary antibodies (TLR4, Bioss, Beijing, China, 1:1000; phospho-p65, Bioss, Beijing, China, 1:1000; p65, Abmart, Shanghai, China, 1:1000; phospho-IκBα, CST, Danvers, MA, USA, 1:1000; IκBα, Affinity, Liyang, China, 1:1000; MyD88, Affinity, Liyang, China, 1:1000; GAPDH, Bioworld, Nanjing, China, 1:5000) at 4 °C. Afterward, the membranes were rinsed with PBS containing 0.1% Tween 20 three times and incubated with goat anti-rabbit secondary antibody (ZSGB Bio., Beijing, China, 1:5000) at 37 °C for 1 h. Ultimately, the protein bands were visualized using enhanced chemiluminescence supersensitive luminescent liquid (Solarbio, Beijing, China) in Amersham Imager 600 (Cytiva, Marlborough, MA, USA), and the gray-scale values were analyzed using ImageJ 8.0 (National Institutes of Health, Bethesda, MD, USA).

### 2.9. Data statistical Analysis

All data were represented as mean ± standard deviation. Differences were determined by one-way ANOVA followed by Tukey’s post hoc test using SPSS 22.0 statistical software (SPSS Inc., Chicago, IL, USA). *p* < 0.05 was considered statistically significant.

## 3. Results

### 3.1. Recombinant Expression of Fusion Protein IFN_OaBac5mini

**Figure 1:**
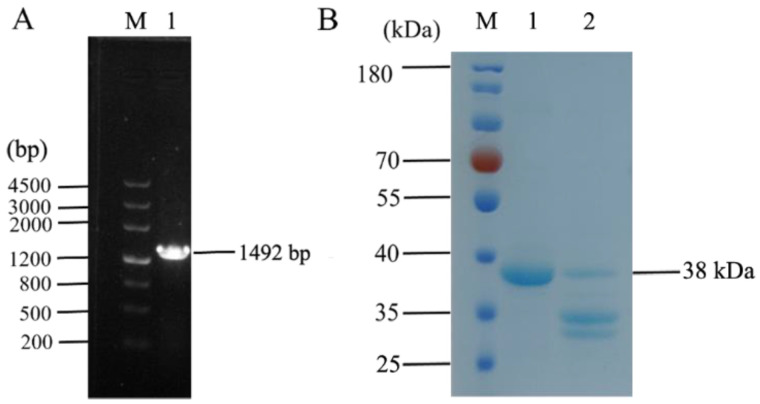
Identification and expression of fusion recombinant vector. (**A**) Identification of recombinant pET-32a::IFN_OaBac5mini using PCR. M: DL4500 DNA Marker, 1: PCR products from ampicillin-resistant strain. (**B**) SDS-PAGE analysis of purified recombinant IFN_OaBac5mini. M: Prestained protein Marker, 1: purified IFN_OaBac5mini, 2: enterokinase-digested IFN_OaBac5mini.

The recombinant expression vector, pET-32a::IFN_OaBac5mini, was constructed, and the length of the PCR products of the positive strains containing recombinant vectors was 1492 bp (Figure 1A). Furthermore, fusion protein IFN_OaBac5mini was generated by induction with IPTG and purification in Ni-NTA column, and recombinant OaBac5mini was obtained by enterokinase digestion (Figure 1B). The yield of recombinant IFN_OaBac5mini was up to 2 mg/mL (BCA kit detection), containing 250 μg/mL OaBac5mini (the molecular weight ratio of IFN to OaBac5mini is 7:1).

### 3.2. Recombinant IFN_OaBac5mini Exhibited no Obvious Cytotoxicity

**Figure 2:**
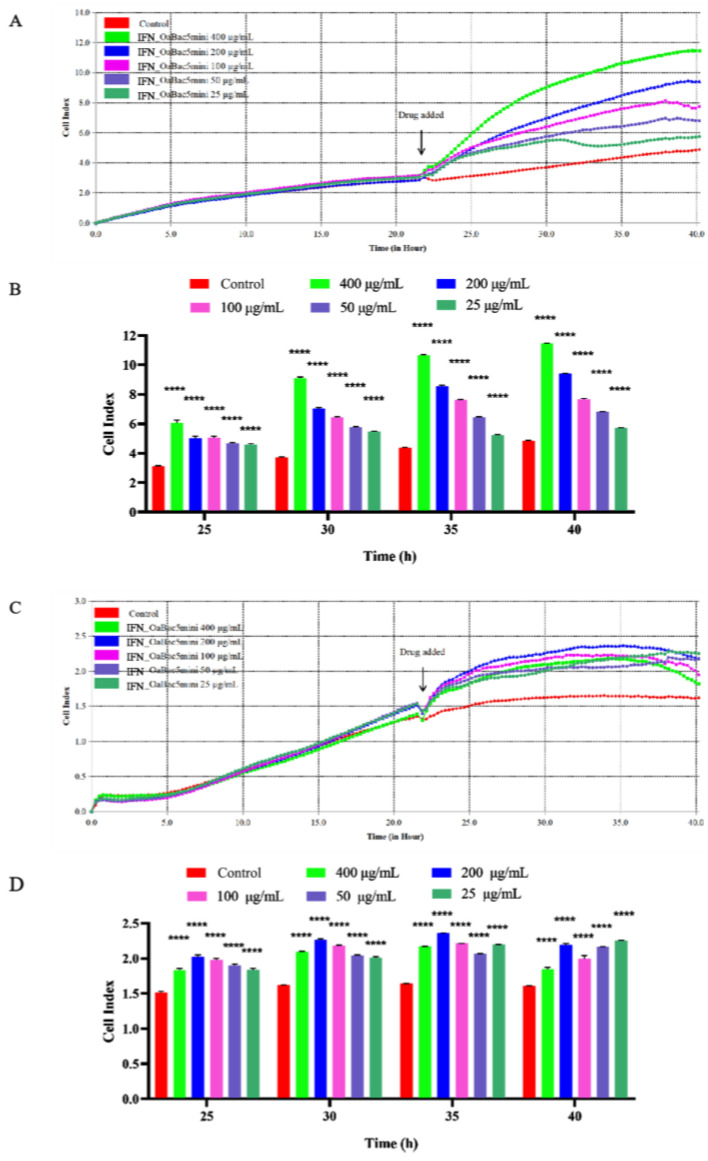
Cytotoxicity of digested IFN_OaBac5mini to IPEC−J2 cells and RAW 264.7 cells. Cell indexes of IPEC−J2 cells (**A**) and RAW 264.7 cells (**C**) treated with different concentrations of IFN_OaBac5mini are shown. Recombinant IFN_OaBac5mini significantly promoted the proliferation of IPCE−J2 cells (**B**) (*p* < 0.0001) and RAW 264.7 cells (**D**) (*p* < 0.0001) without obvious cytotoxicity. **** *p* < 0.0001 vs. control group. *n* = 4.

The cytotoxicity of IFN_OaBac5mini to IPEC-J2 and RAW 264.7 cells was evaluated. RTCA results (Figure 2) showed that the cell indexes of JPEC-J2 and RAW 264.7 cells treated with IFN_OaBac5mini were significantly increased compared to those of the control group (*p* < 0.0001). The results indicated that IFN_OaBac5mini exhibited no cytotoxicity and promoted the proliferation of IPEC-J2 and RAW 264.7 cells.

### 3.3. Recombinant OaBac5mini Alleviated the Drop in the Cell Index of S. Pullorum-Infected Cells

**Figure 3:**
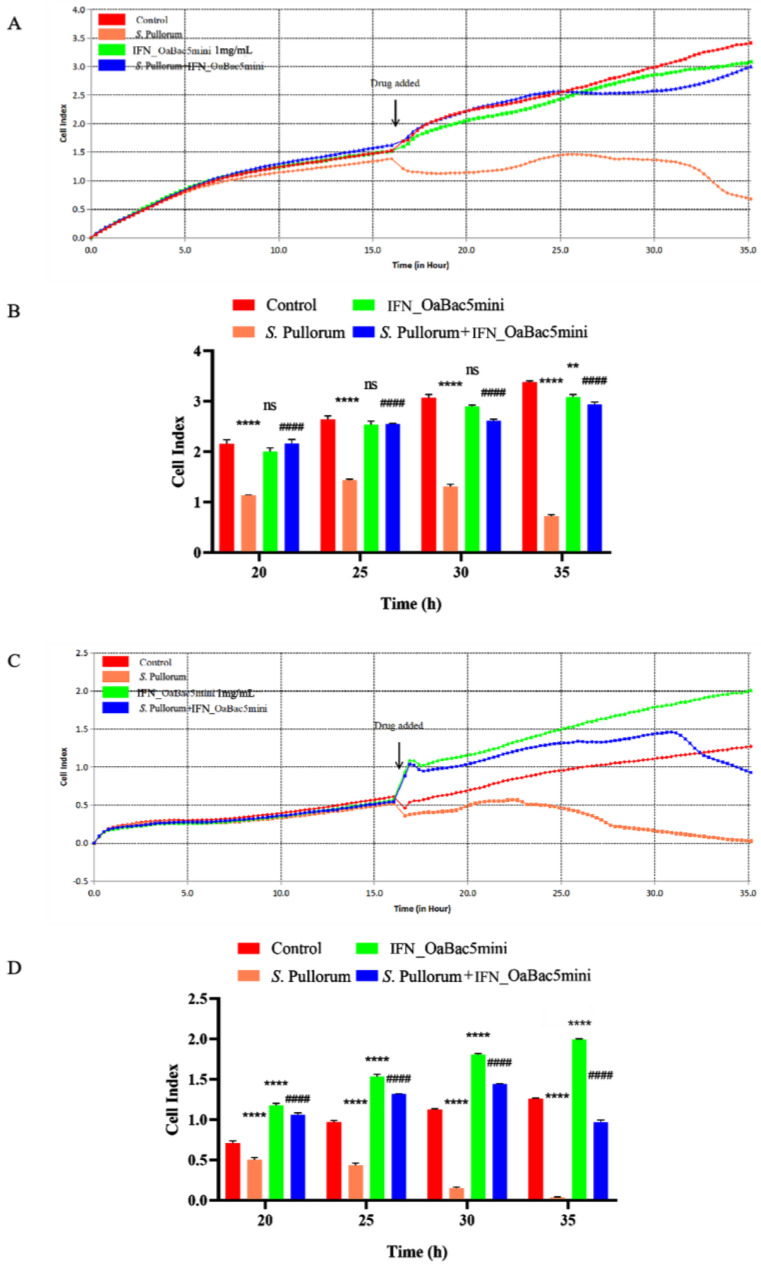
Cell viability of IPEC−J2 and RAW 264.7 cells treated with *S.* Pullorum and/or recombinant OaBac5mini. Cell indexes of IPEC−J2 cells (**A**) and RAW 264.7 cells (**C**) are shown. *S.* Pullorum significantly decreased the cell indexes of IPEC−J2 cells (**B**) (*p* < 0.0001) and RAW 264.7 cells (**D**) (*p* < 0.0001), and recombinant IFN_OaBac5mini significantly increased the cell indexes of IPEC−J2 cells (**B**) (*p* < 0.0001) and RAW 264.7 cells (**D**) (*p* < 0.0001). ns: *p* > 0.05 vs. control group, ** *p* < 0.01 vs. control group, **** *p* < 0.0001 vs. control group, ^####^ *p* < 0.0001 vs. *S.* Pullorum group. *n* = 4.

RTCA was conducted using IPEC-J2 and RAW 264.7 cells treated by *S*. Pullorum with or without recombinant OaBac5mini to real-time evaluate the effect of recombinant OaBac5mini on the viability of *S.* Pullorum-infected cells. Results (Figure 3) showed that *S.* Pullorum significantly decreased the cell indexes of IPCE-J2 cells (Figure 3A,B) and RAW 264.7 cells (Figure 3C,D, *p* < 0.0001) compared with the control group. The cell indexes in the *S*. P + IFN_OaBac5mini group were significantly increased compared with the *S.* Pullorum group (*p* < 0.0001), which indicated that recombinant OaBac5mini alleviated the drop in the cell index of *S.* Pullorum-infected intestinal epithelial cells and macrophages.

### 3.4. Recombinant OaBac5mini Suppressed the mRNA Expression of Pro-Inflammatory Cytokines Induced by S. Pullorum

**Figure 4:**
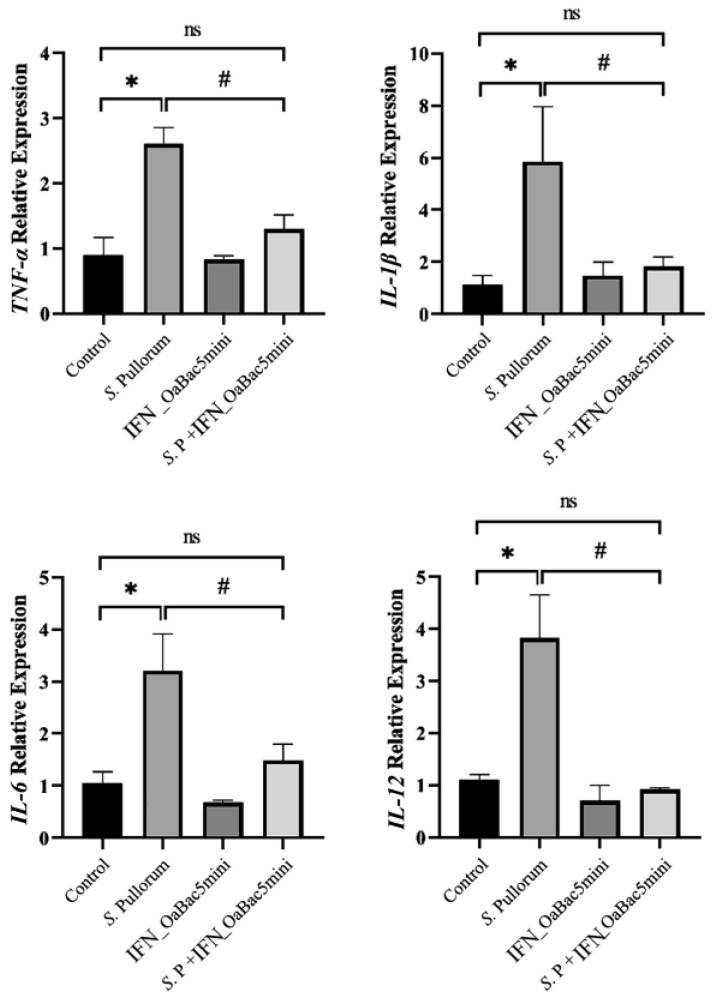
mRNA expression levels of pro-inflammatory cytokines. Relative expression levels of *TNF-α*, *IL-1β*, *IL-6*, and *IL-12* in RAW 264.7 cells were significantly upregulated by *S.* Pullorum (*p* < 0.05). mRNA expression levels of the pro-inflammatory cytokines in the *S*. P + IFN_OaBac5mini group were significantly downregulated compared with those in the *S.* Pullorum group (*p* < 0.05). Data represent results from triplicate experiments. ns: *p* > 0.05 vs. control group, * *p* < 0.05 vs. control group, ^#^ *p* < 0.05 vs. *S.* Pullorum group.

The transcription levels of pro-inflammatory cytokines, including *TNF-α*, *IL-1β*, *IL-6*, and *IL-12*, were significantly upregulated by *S.* Pullorum in IPEC-J2 and RAW 264.7 cells (*p* < 0.05, Figure 4). Furthermore, the mRNA expression levels of such pro-inflammatory factors were significantly downregulated in the *S*. P + IFN_OaBac5mini group compared with the *S.* Pullorum group (*p* < 0.05), but exhibited no obvious changes compared with the control group (*p* > 0.05).

### 3.5. Recombinant OaBac5mini Attenuated the Increase in Organ Indexes in S. Pullorum-Challenged Chicks

**Figure 5:**
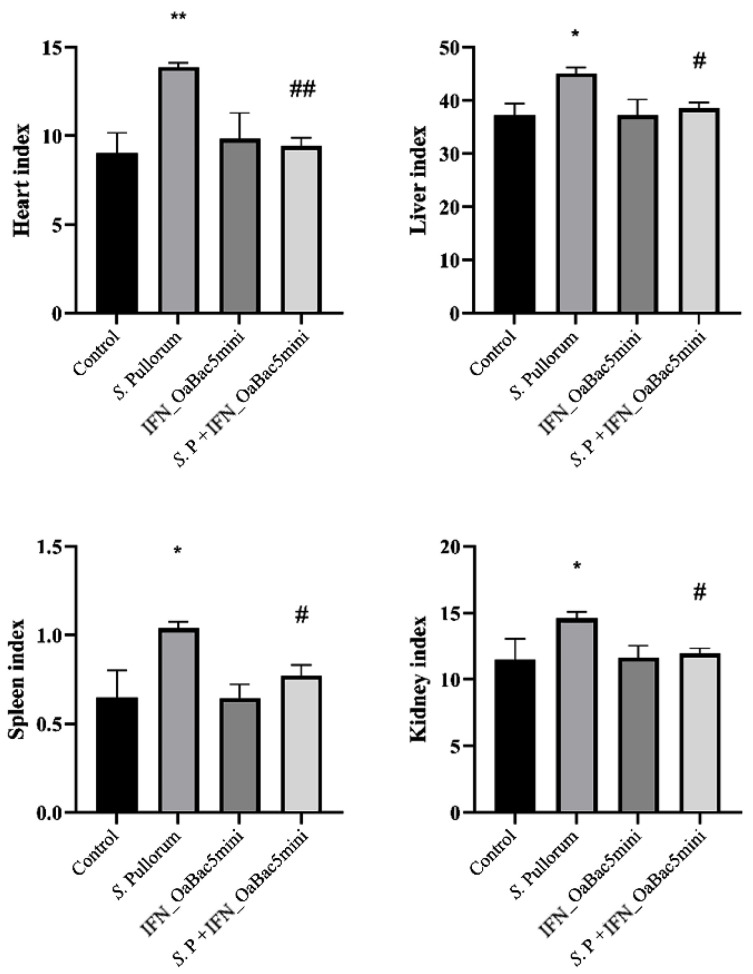
Organ indexes. Organ indexes of the heart, liver, spleen, and kidney of the *S.* Pullorum infection group were significantly increased compared with those of the control group (*p* < 0.05 and *p* < 0.01). Recombinant IFN_OaBac5mini significantly attenuated the increase in organ indexes (*p* < 0.05 and *p* < 0.01). * *p* < 0.05 and ** *p* < 0.01 vs. control group; ^#^ *p* < 0.05 and ^##^ *p* < 0.01 vs. *S.* Pullorum infection group. *n* = 15.

The organ indexes of heart, liver, spleen, and kidney were investigated after the trial. The results (Figure 5) showed that the chicks challenged with *S.* Pullorum exhibited significantly increased organ indexes compared with the control (*p* < 0.05). However, the organ indexes of the IFN_OaBac5mini treated PD chicks were significantly decreased compared with the *S.* Pullorum group (*p* < 0.05), which indicated that recombinant OaBac5mini attenuated the increase in organ indexes triggered by *S.* Pullorum.

### 3.6. Recombinant OaBac5mini Decreased Organ Bacterial Loads in S. Pullorum-Challenged Chicks

**Figure 6:**
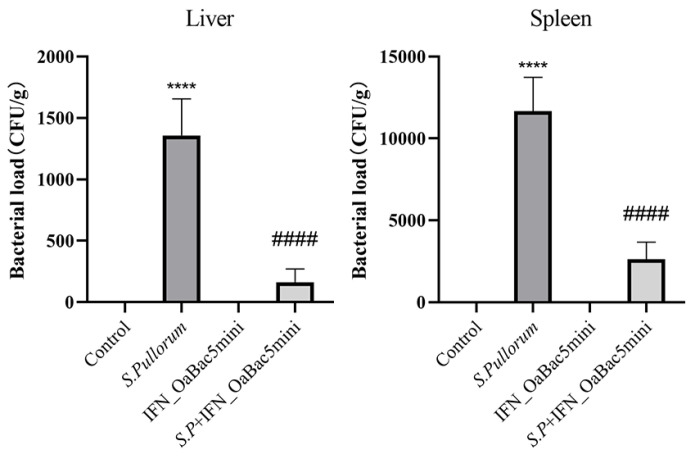
Organ bacterial loads. No bacterial loads were detected in the liver and spleen of the control group and IFN_OaBac5mini treatment group. The bacterial loads of the *S.* Pullorum infection group were significantly increased (*p* < 0.0001), and recombinant IFN_OaBac5mini significantly attenuated the increase (*p* < 0.0001). **** *p* < 0.0001 vs. control group, ^####^ *p* < 0.0001 vs. *S.* Pullorum group. *n* = 15.

The bacterial loads in the liver and spleen were quantified using a *S.* Pullorum selective plate to explore the inhibiting effect of recombinant OaBac5mini on the colonization of *S.* Pullorum in vivo. Results (Figure 6) showed that no bacteria were detected in the control and OaBac5mini group, but the bacterial loads in the liver and spleen of the chicks with PD were significantly increased (*p* < 0.0001). Conversely, the bacterial loads of IFN_OaBac5mini treated PD chicks significantly decreased in the liver and spleen compared with those in the *S.* Pullorum group (*p* < 0.0001).

### 3.7. Recombinant OaBac5mini Ameliorated Histopathological Changes in PD Chicks

**Figure 7:**
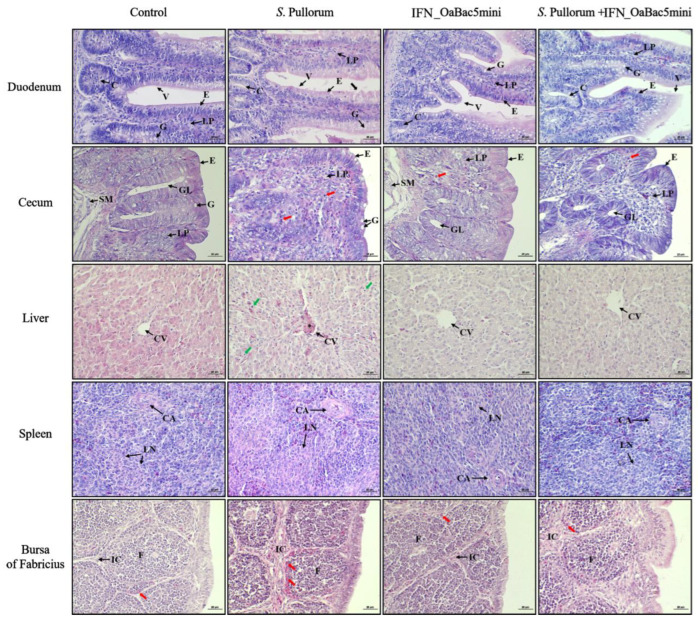
HE staining of the duodenum, cecum, liver, spleen, and bursa of Fabricius slices. Duodenum: black arrow, damaged epithelium; V, villi; C, crypts; E, epithelium; LP, lamina propria; G, goblet cell. Cecum: red arrow, pseudoacidophilic granulocyte; E, epithelium; LP, lamina propria; SM, sub-mucosa; GL, gland; G, goblet cell. Liver: green arrow, macrophages; *, congested central vein; CV, central veins. Spleen: CA, central arteriole; LN, lymphoid nodule. Bursa of Fabricius: red arrow, pseudoacidophilic granulocyte; F, bursal follicule; IC, interfollicular connective tissue.

HE staining of organ slices indicated that *S.* Pullorum triggered histopathological changes and inflammation in multiple organs (Figure 7). Duodenum sections showed that *S.* Pullorum impaired the epithelium of duodenal villi in PD chicks, whereas OaBac5mini prevented the damage, showing intact villi in the *S*. P + IFN_OaBac5mini group. In cecum sections, complete cecum structure with well-developed intestinal glands and a clear epithelial boundary was observed in all groups except for the *S.* Pullorum infection group, which had more goblet cells in the epithelium. Moreover, a large number of pseudoacidophilic granulocytes were observed infiltrating the lamina propria and the interfollicular connective tissue of the bursa of Fabricius in PD chicks. However, the infiltration of pseudoacidophilic granulocytes in the cecum and bursa of Fabricius was alleviated by OaBac5mini. In liver sections, *S.* Pullorum resulted in blood clots that congested in the central vein and macrophage infiltration in hepatic lobules. In the *S*. P + IFN_OaBac5mini group, the hepatic lobule exhibited a complete structure, no blood cells accumulated in the central vein, and the number of macrophages decreased, which was similar than that in the control group. In spleen sections, PD chicks exhibited a thicker lymphatic sheath and a larger lymphoid nodule with more B lymphocytes than other groups, and these changes were alleviated by OaBac5mini treatment.

### 3.8. Recombinant OaBac5mini Modulated the TLR4/MyD88/NF-κB Pathway in PD Chicks

**Figure 8:**
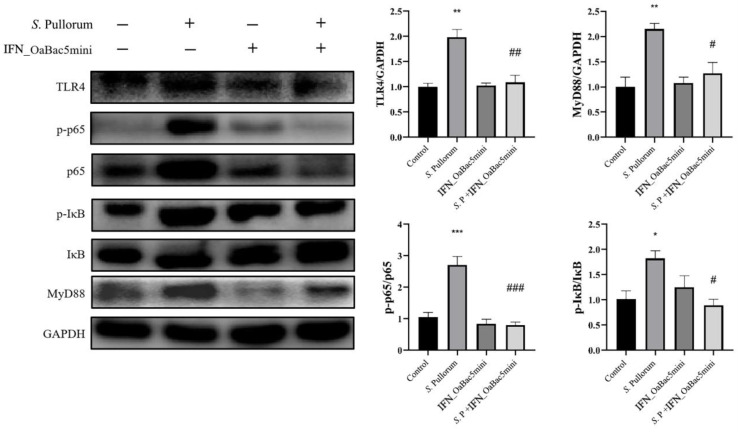
Expression levels of proteins in the TLR4/MyD88/NF-κB pathway in the spleen of chicks. The expression levels were normalized to GAPDH. Data represent results from triplicate experiments. * *p* < 0.05, ** *p* < 0.01, and *** *p* < 0.001 vs. control group. ^#^ *p* < 0.05, ^##^ *p* < 0.01, and ^###^ *p* < 0.001 vs. *S.* Pullorum infection group.

TLR4 is an innate immune receptor that responds to the lipopolysaccharide (LPS) of bacteria and activates NF-κB through a MyD88-dependent pathway. The protein expression levels of the TLR4/MyD88/NF-κB pathway were investigated to explore the mechanism of recombinant OaBac5mini in suppressing the inflammation induced by *S.* Pullorum in PD chicks. Western blot results (Figure 8) showed that *S.* Pullorum activated the TLR4/MyD88/NF-κB pathway, in which the protein expression levels of TLR4 and MyD88, the ratio of p-p65 to p65, and the ratio of p-IκB to IκB were significantly upregulated (*p* < 0.05). The recombinant OaBac5mini significantly downregulated the expression and ratio of such proteins (*p* < 0.05) compared with the *S.* Pullorum infection group, which indicated that recombinant OaBac5mini effectively suppressed the activation of the TLR4/MyD88/NF-κB pathway in PD chicks.

## 4. Discussion

Antibiotics used as feed supplements for growth promotion are restricted in several countries because of the rapid spread of drug-resistant and multidrug-resistant bacterial pathogens [8,11], leading to an urgent need to find new antibiotic alternatives and their suitable production methods. AMPs are important components of animals’ innate immune systems and are promising as next-generation antibiotics owing to their broad antimicrobial spectrum [23,24]. AMP OaBac5mini, which exhibits strong antibacterial activity against Gram-negative bacteria and has good stability and low cytotoxicity [16,17], is worthy of further study. However, the artificial genetic engineering production of OaBac5mini has not been reported. The recombinant approach offers the most cost-effective means for large-scale peptide manufacture, and *E. coli* is the most widely used recombinant expression system with high expression yields [18]. However, it poses serious challenges because AMP has a molecular weight of less than 50 amino acid residues and is toxic to the host cells of *E. coli* [18]. Therefore, we constructed the fusion expression system, pET-32a::IFN_OaBac5mini, which combined IFN and OaBac5mini with an enterokinase site (Figure S1) to overcome these disadvantages. After the culture and induction conditions and purification methods were optimized, the purified fusion protein reached an expression yield of up to 2 mg/mL containing 250 μg/mL recombinant OaBac5mini (BCA kit detection results), and it exhibits similar or better antibacterial activity against *S*. Pullorum compared to traditional antibiotics (Table S1), with negligible cytotoxicity to small intestinal epithelial cells and macrophages (Figure 2).

In countries with developing poultry industries, PD is the most consequential poultry disease caused by *S.* Pullorum, which poses considerable economic costs to poultry producers [25]. *S.* Pullorum causes severe septicemia in young birds and can colonize the reproductive tract, resulting in transovarial transmission to eggs (vertical transmission) [26]. In addition, *S.* Pullorum from contaminated environments (horizontal transmission) invades the intestinal tract by colonizing and proliferating in intestinal epithelial cells and further proliferates in macrophages or dendritic cells by phagocytosis through interrupting intestinal epithelium [27], where it can directly diffuse and translocate to the liver and spleen through blood circulation [28]. In the present study, *S.* Pullorum damaged the intestinal villous epithelium and was isolated from the liver and spleen of the PD chicks, but recombinant OaBac5mini ameliorated the histopathological changes of the intestine and considerably decreased the bacterial loads in the liver and spleen (Figure 6 and Figure 7), which indicated that OaBac5mini blocks the translocation of *S.* Pullorum.

*S.* Pullorum can evidently stimulate host immunity. Setta et al. [29] reported that *Salmonella* infection increased the number of B-lymphocytes and macrophages in the cecal tonsils of infected birds. In this study, histopathological examination indicated that PD chicks presented an increase in macrophages and B-lymphocytes in the liver and spleen and infiltration of pseudoacidophilic granulocytes in the cecum and bursa of Fabricius (Figure 7). Recombinant OaBac5mini probably attenuated the increase of such innate immune cells by decreasing the number of infiltrating *S.* Pullorum through blocking the translocation of *S.* Pullorum.

Such inflammatory cell infiltration in the histological section implies the immune stimulation and inflammatory cell chemotaxis caused by *S.* Pullorum. Toll-like receptors (TLRs) are key elements of innate immunity against invading pathogens and activate a variety of host defense signal pathways [30]. TLR4 responds to the LPS secreted by Gram-negative bacteria; activates NF-κB through a MyD88-dependent pathway [31]; and finally, induces the expression of genes related to immune stimulation, cell apoptosis, and inflammatory cell chemotaxis [32]. Li et al. [33] demonstrated that the TLR4/MyD88-dependent pathway plays a role in *S.* Pullorum-infected chicks. In the present study, *S.* Pullorum upregulated the expression of TLR4 and MyD88 (Figure 8), which indicated that *S.* Pullorum activated the TLR4/MyD88/NF-κB pathway. The binding of IκB to the NF-κB subunit results in the conformational changes of NF-κB, which inhibits the binding of NF-κB p65 to the target gene [34]. When phosphorylated IκB is degraded by ubiquitination, p65 is released into the nucleus and is phosphorated to regulate the transcription of pro-inflammatory cytokines TNF-α, IL-1β, IL-6, and IL-12 [35]. In the present study, *S.* Pullorum increased the ratio of p-p65 to p65 and the ratio of p-IκB to IκB, as well as inducing the upregulation of the expression of the pro-inflammatory cytokines, *TNF-α*, *IL-1β*, *IL-6*, and *IL-12*. The results indicated that the innate immune performance of PD chicks was severely impaired by *S.* Pullorum through the TLR4 pathway. AMPs, as effector molecules, are important for innate immunity. The body expresses AMPs to play a corresponding immunomodulatory effect against foreign bacterial infection [36]. Shin et al. and Sun et al. reported that AMPs are capable of tackling inflammation by targeting TLR signaling to downregulate the expression of pro-inflammatory mediators [37,38]. In the current study, recombinant OaBac5mini blocked the increased expression of related proteins in the TLR4/MyD88/NF-κB pathway (Figure 8) and inhibited both the phosphorylation of NF-κB p65 and the transcription of its pro-inflammation target genes, *TNF-α*, *IL-1β*, *IL-6*, and *IL-12*, in PD chicks. The results implied that OaBac5mini modulated the innate immunity impaired by *S.* Pullorum. OaBac5 belongs to the cathelicidin family, and Niyonsaba et al. and Mookherjee et al. reported that LL-37, another cathelicidin family member, can prevent the translocation of the NF-κB subunit p65 or directly bind to LPS, which carries negative charges, preventing its interaction with LPS-binding protein and therefore inhibiting the activation of TLR4 and downstream signaling pathways [39,40]. OaBac5mini has eight positive charges [16], which is more than the six positive charges of LL-37 [41]. This finding suggests that OaBac5 may modulate the TLR4 pathway by reducing the amount of LPS or by binding to LPS. In short, recombinant OaBac5mini alleviated inflammatory response by modulating innate immunity through the TLR4/MyD88/NF-κB pathway in PD chicks.

## 5. Conclusions

Fusion protein IFN_OaBac5mini was expressed by the *E. coli* recombinant protein expression system and exhibited good antibacterial activity against *S.* Pullorum in vitro and in vivo with no obvious cytotoxicity. Recombinant OaBac5mini inhibited the colonization of *S.* Pullorum, attenuated the organomegaly and histopathological changes of PD chicks, and ameliorated inflammation response through the TLR4/MyD88/NF-κB pathway.

## Data Availability

The data sets generated or analyzed during the present study are available from the corresponding author upon request.

## Supplementary Materials

The following supporting information can be downloaded at: https://www.mdpi.com/article/10.3390/ani13091515/s1, Figure S1 Construction strategy for recombinant expression vector pET-32a::IFN_OaBac5mini. IFN_OaBac5mini fragment was amplified by three steps PCR using pET 28a-SUMO:IFN_PG as original template. IFN_OaBac5mini fragment (612 bp) and pET-32a (+) plasmid were digested with *Bam*HI and *Xho*I, purified and recombined to construct the recombinant expression vector; Table S1 MICs of different antibiotic agents against S. Pullorum CVCC 530 (unit: μg/mL).
