# Supplementary material for: Recombinant Antimicrobial Peptide OaBac5mini Alleviates Inflammation in Pullorum Disease Chicks by Modulating TLR4/MyD88/NF-κB Pathway
Source: Animals (Basel). 2023 Apr 30;13(9):1515. doi: 10.3390/ani13091515 (PMC10177235; doi:10.3390/ani13091515)
Supplement: Supplementary file 1 [file animals-13-01515-s001.zip › animals-2318249-supplementary.pdf]

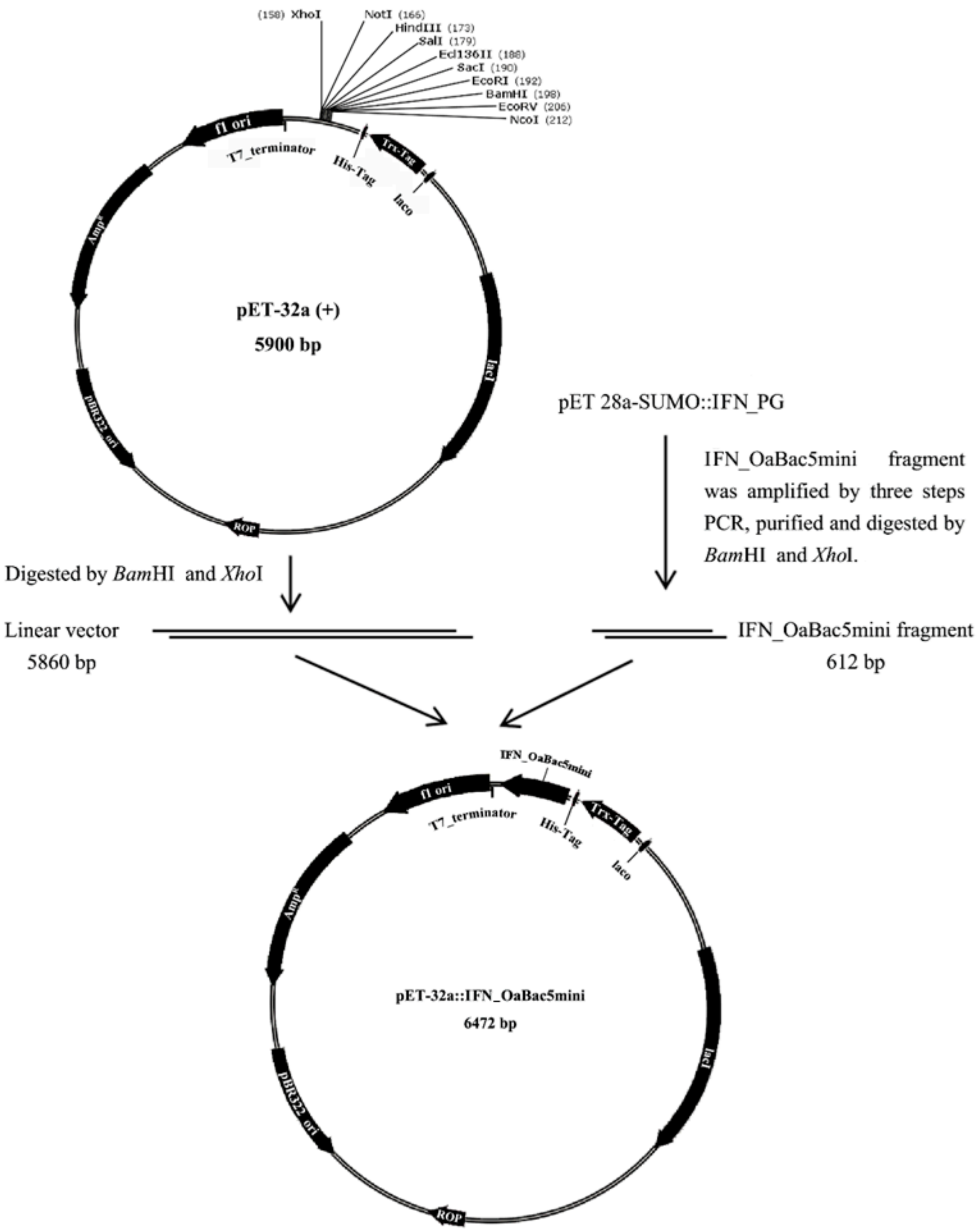

Figure S1 Construction strategy for recombinant expression vector pET-32a::IFN_OaBac5mini. IFN_OaBac5mini fragment was amplified by three steps PCR using pET 28a-SUMO:IFN_PG as original template. IFN_OaBac5mini fragment (612 bp) and pET-32a (+) plasmid were digested with *Bam*HI and *Xho*I, purified and recombined to construct the recombinant expression vector.

Table S1 MICs of different antibiotic agents against *S*. Pullorum CVCC 530 (unit: μg/mL)

Antibacterial agents	MICs
florfenicol	4
kanamycin	2.67
tetracycline	53.33
OaBac5mini	1.56
